# Cybervictimization and Adolescent Internet Addiction: A Moderated Mediation Model

**DOI:** 10.3390/ijerph18052427

**Published:** 2021-03-02

**Authors:** Mucheng Xin, Pei Chen, Qiao Liang, Chengfu Yu, Shuangju Zhen, Wei Zhang

**Affiliations:** 1 Department of Psychology and Research Center of Adolescent Psychology and Behavior, School of Education, Guangzhou University, Guangzhou 510006, China; 2111908157@e.gzhu.edu.cn (M.X.); a13430205519@163.com (P.C.); 2111808166@e.gzhu.edu.cn (Q.L.); 2 School of Psychology and Center for Studies of Psychological Application, South China Normal University, Guangzhou 510631, China; shuangjuzhen@gmail.com

**Keywords:** adolescent, cybervictimization, internet addiction, rejection sensitivity, parent–adolescent communication

## Abstract

Previous research indicates that cybervictimization can lead to adolescent Internet addiction; however, there is a gap in the knowledge about the mediating and moderating variables facilitating this relationship. This study examines the role of rejection sensitivity as a mediator in this relationship and the role of parent–adolescent communication as a moderator for this mediation effect among Chinese adolescents. Participants were 1006 adolescents (M = 13.16 years, SD = 0.67), who completed the questionnaires anonymously. The questionnaires assessed the four variables of interest. Descriptive statistics and structural equation modeling were used for data analysis. The results show that the positive association between cybervictimization and adolescent Internet addiction is mediated by rejection sensitivity. Moreover, this indirect effect is stronger for adolescents with low parent–adolescent communication than for those with high parent–adolescent communication.

## 1. Introduction

Internet addiction refers to the uncontrollable, excessive, and compulsive use of the Internet, which has recently become a concern due to its psychological and physiological effects [1,2]. According to the latest report on the use of Internet among Chinese people by the China Internet Network Information Center in December of 2020 [3], Internet users in China have reached 989 million, of which 13.5% were aged 10–19; this data report suggested the importance of providing guidance to adolescents about appropriate Internet usage and prevention of Internet addiction. Increasing amount of evidence has demonstrated that Internet addiction could lead to negative outcomes, such as poor academic adjustment [4] and mental health issues such as depressive symptoms [5,6]. Therefore, it is necessary to explore the key mechanisms and pathways of adolescent Internet addiction development to provide valuable information for the continued development and refinement of effective prevention strategies and early intervention initiatives in the future.

A recent study suggested an increasing prevalence of cybervictimization, and the rate of cybervictimization is 23.8% for China [7,8]. The effect of cybervictimization on adolescent Internet addiction has attracted an increasing amount of attention [9,10]. Cybervictimization is a common form of online harassment among adolescents. It refers to the intentional and repetitive harm caused via various modes of online communication. According to the self-medication hypothesis, self-medication motivation is one of the most compelling reasons for addiction instead of simply escaping from reality [11]. Individuals who experienced cybervictimization tend to have difficulty dealing with their emotions and relationships [12]. Therefore, individuals who have experienced cybervictimization are more likely to experience Internet addiction to reduce their emotional tension and stress [7]. Consistent with this perspective, addictive behaviors could be a behavioral response to existing pressures, such as emotional distress and interpersonal stress [13]. Specifically, Internet addiction can be considered a coping strategy to escape from interpersonal stress causing by cybervictimization [9]. Additionally, adolescents who experience higher levels of cybervictimization have been shown to be socially isolated and lacking in social support; they attempt to compensate for these social deficits by engaging in Internet overuse [14]. Although previous studies have primarily focused on the direct link between cybervictimization and Internet addiction [9,13], there is very little literature on the potential variables that mediate and/or moderate this association directly/indirectly. To address these gaps, we gathered data from a sample of Chinese adolescents to examine the mediating effect of rejection sensitivity and the moderating effect of parent–adolescent communication.

### 1.1. Rejection Sensitivity as a Mediator

Rejection sensitivity is defined as the cognitive and emotional tendency to anticipate, perceive, and overreact defensively to the signs of rejection by others [15]. According to the rejection sensitivity model [16], adolescents who suffer from interpersonal rejection tend to experience higher rejection sensitivity and then lead to maladaptive behavioral responses [17,18]. That is to say, rejection sensitivity might mediate the relationship between cybervictimization and Internet addiction.

There are several reasons to believe that cybervictimization can promote rejection sensitivity. First, Downey and Feldman [15] demonstrated that the experience of rejection could develop into some form of “basic mistrust” about interpersonal relationships, which leads to higher rejection sensitivity [19,20]. Second, although little study has examined the impact of cybervictimization on rejection sensitivity directedly [17], several studies have indicated that adolescents that often suffer from cyberbullying develop a low social self-perception and make a negative evaluation of their own social ability, which leads to the development of social anxiety [20,21]. At the same time, it has been shown that social anxiety is associated with greater rejection sensitivity [22]. Thus, it is reasonable that cybervictimization could promote rejection sensitivity.

Moreover, adolescents with higher levels of rejection sensitivity are more likely to develop Internet addiction [23]. A prior study has shown that the Internet may act as an adaptive coping strategy to regulate negative emotions and decrease rejection stress in virtual environments [23,24,25,26]. For example, Farahani et al. [24] found that Internet usage (such as social media platforms or video games) can improve the subjective perception of the individual with high rejection sensitivity. The time of online Internet use due to the excessive focus on the virtual world may increase, which may contribute to the risk of Internet addiction development among adolescents. Therefore, rejection sensitivity could potentially be an underlying mediating mechanism between cybervictimization and Internet addiction. Thus, we propose the following hypothesis:

**Hypothesis** **1.** *Rejection sensitivity mediates the relationship between cybervictimization and adolescent Internet addiction*.

### 1.2. Parent–Adolescent Communication as a Moderator

Although it is possible that cybervictimization increases the risk of Internet addiction development via rejection sensitivity, not all adolescents who suffer from cybervictimization experience an equivalent trajectory of rejection sensitivity and Internet addiction. This variation may be caused by protective factors such as parent–adolescent communication, which may moderate the impact of adversities on adjustment problems [27]. Parent–adolescent communication is a process where parents and adolescents share their attitudes, values, knowledge, and expectations with each other. Adolescents spend most of their time with their parents; therefore, they typically have many opportunities to communicate with their children daily. Thus, parents play an important role in adolescents’ development [28]. Research evidence has demonstrated that Chinese adolescents who experienced more positive parent–adolescent communication were less likely to suffer from Internet addiction [29,30]. For instance, Liu et al. [30] examined the predictive role of parent–adolescent communication on Internet addiction and found that strong parent–adolescent communication can improve adolescents’ understanding of their immediate emotional reactions and allow them to recognize their emotions, rather than rely on the Internet alone, which would help improve their adaptability. Another recent study found that positive parent–adolescent communication was positively associated with the level of youths’ subjective well-being and negatively associated with rejection sensitivity [31,32]. Specifically, adolescents with parental support and strong family relationships tend to initiate conversations with their parents, instead of showing high rejection sensitivity after being cyberbullied, thereby lessening the impact of negative events [33,34,35]. 

According to the self-determination theory, there are several ways families can satisfy most of the adolescent basic psychological needs, such as the need for autonomy, competence, and relatedness [36], which can ease the negative impact of adverse experiences on adolescents’ adjustment. First, positive parent–adolescent communication is characterized by a high level of parental involvement and support. This warm and intimate relationship can compensate for the need to belong of adolescents who are frustrated after experiencing cybervictimization [37]. Second, parent–adolescent communication can provide problem-solving skills for adolescents and enable them to face challenges and deal with problems [30,38]. Additionally, parents help and guide adolescents with rejection experience through discussing and explaining rejection, which impacts youths’ understanding and minimizes attributions of these rejection experiences as internal (self-related) [39]. Thus, when adolescents with positive parent–adolescent communication experience cybervictimization, they may deal with the problem in several ways other than using the Internet as an avoidance coping strategy. Furthermore, previous studies have also highlighted the moderating role of parent–adolescent communication in reducing the impact of environmental risk. For instance, it has been shown that higher levels of parent–adolescent communication can promote a positive response to negative life events among the youth, which acts as a buffer for adolescents against the negative influence of bullying [40]. Similarly, it has been found that active parent–adolescent communication towards the negative consequences of Internet reliance can reduce the levels of pathological use of the Internet [41]. Therefore, we proposed the following hypothesis:

**Hypothesis** **2.** *Parent–adolescent communication can moderate the indirect relationship between cybervictimization and Internet addiction. Specifically, adolescents with higher levels of positive parent–adolescent communication (compared to lower) would be less likely to show rejection sensitivity in the context of cybervictimization and would experience a lower severity of Internet addiction in the face of rejection sensitivity than those with low levels of positive parent–adolescent communication*.

### 1.3. The Present Study

**Figure 1:**
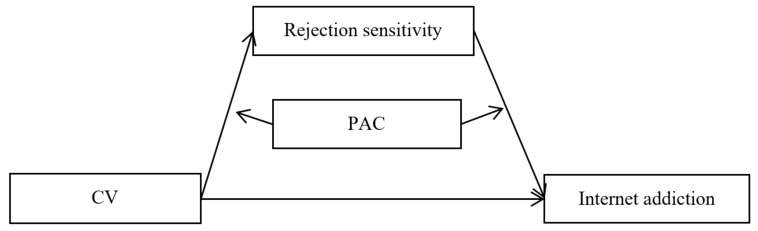
The proposed mediated moderation model. Note: CV = Cybervictimization, PAC = parent–adolescent communication.

Recent studies have examined the mechanisms underlying the association between cybervictimization and Internet addiction among Chinese adolescents. However, there are still several major questions that remain unanswered:

(a) Does rejection sensitivity mediate the relationship between cybervictimization and adolescent Internet addiction?
(b) Does parent–adolescent communication act as a buffer for the mediating effect of rejection sensitivity in the relationship between cybervictimization and Internet addiction? (Figure 1).

We hypothesized that a higher severity of cybervictimization is associated with stronger rejection sensitivity, which in turn is a risk factor for Internet addiction. Moreover, this mediation link was hypothesized to be moderated by parent–adolescent communication. The indirect pathways in the mediation link are much weaker for adolescents who have better communication with their parents.

## 2. Method

### 2.1. Participant

Participants were 1006 Chinese adolescents recruited from three junior middle schools in Guangdong province, southern China. Questionnaires were completed anonymously. The gender distribution of the participants in this study was 48.20% males (*n* = 485) and 51.80% females (*n* = 521). The participants ranging from 12 to 15 years old, and the average age was 13.16 years old (SD = 0.67 years old). See Table 1.

### 2.2. Measures

#### 2.2.1. Cybervictimization

Cybervictimization was assessed using the Cyber Bullying Inventory (CBI) [42]. In this study, we only used the cybervictimization subscale for testing. This questionnaire comprises 18 items assessing the frequency of cybervictimization experienced by the participant in the past 6 months (e.g., “Someone spread rumors about me online”). Items were rated on a 5-point Likert-type response scale ranging from 0 (“Never”) to 4 (“More than five times”). Answers of occasional and above were considered to have been subjected to cybervictimization. A cybervictim is anyone that has been victimized in any of the 18 items. In the present study, 63.22% of youths from the sample were found to be cybervictims. In this study, the Cronbach’s α for this scale was 0.82.

#### 2.2.2. Internet Addiction

The Internet addiction questionnaire was adapted from the Internet gaming disorder questionnaire of nine items compiled by Pontes and Griffiths [43]. It comprised nine items assessing the prevalence of Internet addiction symptoms among the participants in the past 6 months (e.g., “Have you spent more time surfing the Internet than was planned?”). Each item is rated on a 3-point Likert-type response scale, with the options of 0 (“none”), 0.5 (“sometimes”), and 1 (“often”). An average score is calculated across all items, where higher scores reflect greater severity of Internet addiction symptoms. In this study, the Cronbach’s α for this scale was 0.74. 

#### 2.2.3. Rejection Sensitivity

Rejection sensitivity was measured using the rejection sensitivity questionnaire [44], adapted from the rejection sensitivity questionnaire by Downey and Feldman [15]. This questionnaire consisted of 18 items assessing the feelings related to interpersonal experiences (e.g., “I’ve always been afraid of letting people down”), and six of these are scored in reverse. Adolescents rated items on a 5-point Likert-type response scale ranging from 1 (“not at all true”) to 5 (“always true”). Scores were averaged across the 18 items, with higher scores indicating higher levels of rejection sensitivity. The Cronbach’s α for the present sample was 0.86.

#### 2.2.4. Parent–Adolescent Communication

Parent–adolescent communication was measured using the Chinese version of the Parent–adolescent communication questionnaire [45]. This questionnaire consisted of 10 items assessing the frequency of adolescents’ communication with their parents such as daily life, academics, interpersonal interaction, safety, and emotional issues. Adolescents rated items on a 3-point Likert-type response scale ranging from 1 (“never”) to 3 (“often”). Scores were averaged across all items, with higher scores indicating higher levels of communication with parents. Cronbach’s α was 0.68 in this study. 

#### 2.2.5. Control Variables

As adolescents’ age, gender and impulsivity are significant influencing factors in IA [46,47,48], we controlled for these variables in the statistical analyses. Impulsivity was measured by the UPPS-P Scale [49]. Participants rated items on a 4-point Likert-type response scale ranging from 1 (“strongly disagree”) to 4 (strongly agree). Scores were averaged across all items, with higher scores indicating higher impulsivity. Goodness-of-fit for the model was assessed using several standardized indices, including χ^2^/df = 4.85, CFI= 0.90, RMSEA = 0.062. During this time, the Cronbach’s α was 0.82 in this study.

### 2.3. Procedure

Data were collected from participants during school hours by well-trained psychology professors and graduate students. Written informed consent was obtained from participants, teachers, and parents before data collection. Participants were provided with a complete description of the study, and they were instructed to complete the questionnaire independently. To encourage honest reporting, the questionnaires were kept anonymous, and adolescents were given approximately 30 min to complete them. This study was approved by the research ethics committee of the author’s university. 

### 2.4. Statistical Analyses

SPSS (Version 20.0), was used for analyzing descriptive statistics. Furthermore, structural equation modeling was conducted using Mplus 7.1, to examine the mediating and moderating effects of the variables [50]. Goodness-of-fit for the model was assessed using several standardized indices, including *χ*^2^/*df* < 5, CFI > 0.90, RMSEA < 0.08, and SRMR < 0.09 [51].

## 3. Results

### 3.1. Preliminary Analyses

The descriptive statistics and correlation coefficients for all variables are presented in Table 2. The average age of the participants was 13.16 years (SD = 0.67 years). The results indicated that cybervictimization is positively correlated with rejection sensitivity (*r* = 0.23, *p* < 0.01) and Internet addiction (*r* = 0.30, *p* < 0.01), and rejection sensitivity is positively correlated with Internet addiction (*r* = 0.29, *p* < 0.01). Additionally, parent–adolescent communication is negatively correlated with cybervictimization (*r* = −0.14, *p* < 0.01), rejection sensitivity (*r* = −0.13, *p* < 0.01) and Internet addiction (*r* = −0.20, *p* < 0.01). According to Cohen’s (1992) standard [52], the correlation correlations among the constructs are weak, which suggests that parent–adolescent communication may be a moderating mechanism (i.e., when the relationship of “cybervictimization-rejection sensitivity-Internet addiction” occurs, when it is strong and when it is weak). 

### 3.2. Testing for Mediation Effect of Rejection Sensitivity

**Figure 2:**
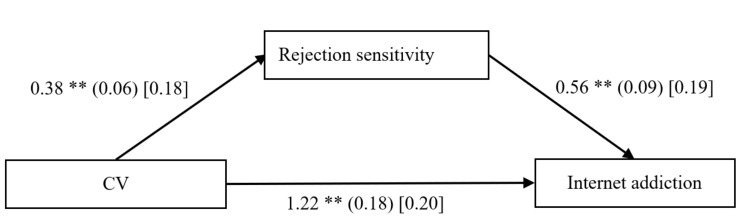
Mediation model of rejection sensitivity between cybervictimization and Internet addiction. Note: CV = cybervictimization. The values outside the brackets are unstandardized coefficients, those in parentheses are standard errors, and those in brackets are standardized coefficients. Paths between gender, age, impulsivity, and each of the variables in the model are not displayed. Of those paths, the following were significant: gender (*b* = −0.18, *SE* = 0.02, *β* = −0.41, *t* = −7.06, *p* < 0.01, 95% CI [−0.22, −0.13]), and impulsivity (*b* = 0.29, *SE* = 0.03, *β* = 0.27, *t* = 9.29, *p* < 0.01, 95% CI [0.23, 0.36]) to rejection sensitivity; gender (*b* = 0.26, *SE* = 0.07, *β* = 0.21, *t* = 3.60, *p* < 0.01, 95% CI [0.12, 0.40]), and impulsivity (*b* = 0.79, *SE* = 0.09, *β* = 0.25, *t* = 8.49, *p* < 0.01, 95% CI [0.61, 0.98]) to Internet addiction. ** *p* < 0.01.

The mediation model presented in Figure 2 revealed an excellent fit to the data (*χ*^2^/*df* = 0.19, CFI = 1.00, RMSEA = 0.00, and SRMR = 0.01). After controlling for gender, age, and impulsivity, it was found that cybervictimization significantly predicted rejection sensitivity (*β* = 0.18, *p* < 0.01, 95% confidence interval [CI]: 0.26–0.50), and rejection sensitivity significantly predicted Internet addiction (*β* = 0.19, *p* < 0.01, 95% CI: 0.38–0.73). Moreover, the residual effect of cybervictimization on Internet addiction was significant (*β* = 0.20, *p* < 0.01, 95% CI: 0.86–1.57). Bootstrapping analyses (number of bootstrap samples = 1000) indicated that rejection sensitivity significantly mediated the relationship between cybervictimization and adolescent Internet addiction (indirect effect = 0.21, 95% CI: 0.12–0.33).

### 3.3. Testing for Moderated Mediation

**Figure 3:**
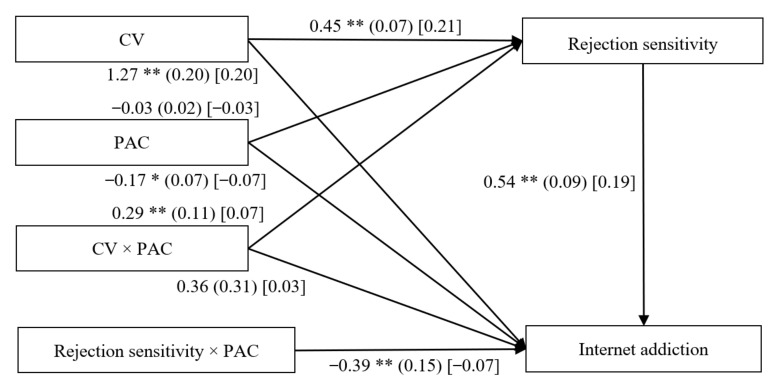
Model of the moderating role of parent–adolescent communication on the indirect relationship between Cybervictimization and Internet addiction. Note: CV = cybervictimization, PAC = parent–adolescent communication. The values outside the brackets are unstandardized coefficients, those in parentheses are standard errors, and those in brackets are standardized coefficients. Paths between gender, age, impulsivity, and each of the variables in the model are not displayed. Of those paths, the following were significant: gender (*b* = −0.18, *SE* = 0.02, β = −0.41, *t* = −7.23, *p* < 0.01, 95% CI [−0.23, −0.13]), and impulsivity (*b* = 0.28, *SE* = 0.03, *β* = 0.26, *t* = 8.29, *p* < 0.01, 95% CI [0.21, 0.34]) to rejection sensitivity; gender (*b* = 0.26, *SE* = 0.07, *β* = 0.21, *t* = 3.59, *p* < 0.01, 95% CI [0.12, 0.40]), and impulsivity (*b* = 0.74, *SE* = 0.10, *β* = 0.24, *t* = 7.63, *p* < 0.01, 95% CI [0.55, 0.93]) to Internet addiction. * *p* < 0.05, ** *p* < 0.01.

The moderated mediation model presented in Figure 3 revealed a good fit to the data (*χ*^2^/*df* = 1.96, CFI = 0.96, RMSEA = 0.04, and SRMR = 0.03). After controlling for gender, age, and impulsivity, it was found that parent–adolescent communication moderated the association between cybervictimization and rejection sensitivity (*β* *=* 0.07, *p* < 0.01, 95% CI: 0.09, 0.50) and between rejection sensitivity and Internet addiction (*β* = 0.07, *p* < 0.01, 95% CI: −0.69, −0.10), respectively. However, the interaction between cybervictimization and parent–adolescent communication in predicting Internet addiction was not significant. Moreover, cybervictimization showed a significant positive association with rejection sensitivity (*β* = 0.21, *p* < 0.01, 95% CI: 0.32, 0.59) and Internet addiction (*β* = 0.20, *p* < 0.01, 95% CI: 0.88, 1.66). However, the predictive effects of parent–adolescent communication on rejection sensitivity and Internet addiction were not significant.

**Figure 4:**
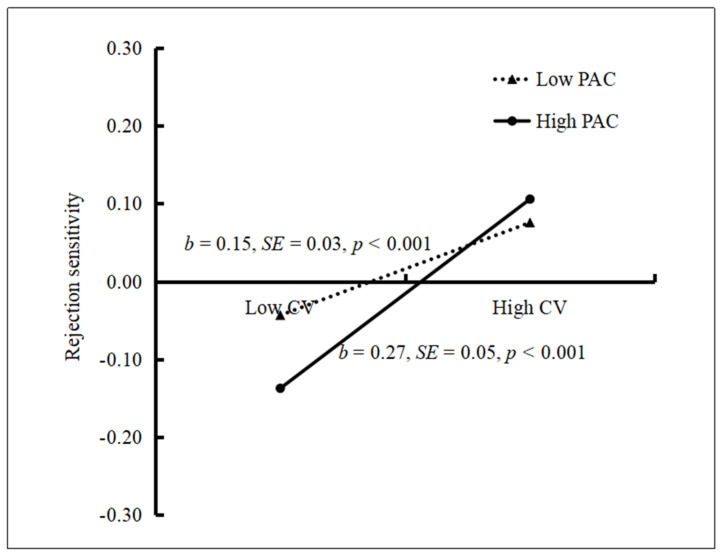
Rejection sensitivity among adolescents as a function of cybervictimization and parent–adolescent communication. Note: CV = cybervictimization, PAC = parent–adolescent communication.

We conducted a simple slope test to assess the moderating effect of parent–adolescent communication between cybervictimization and Internet addiction. It revealed that the positive association between cybervictimization and Internet addiction was much stronger for adolescents with higher parent–adolescent communication (*t* *=* 5.78, *p* < 0.01, 95% CI: 0.40, 0.82) than for those with lower parent–adolescent communication (*t* = 4.28, *p* *<* 0.01, 95% CI: 0.16, 0.43). See Figure 4.

**Figure 5:**
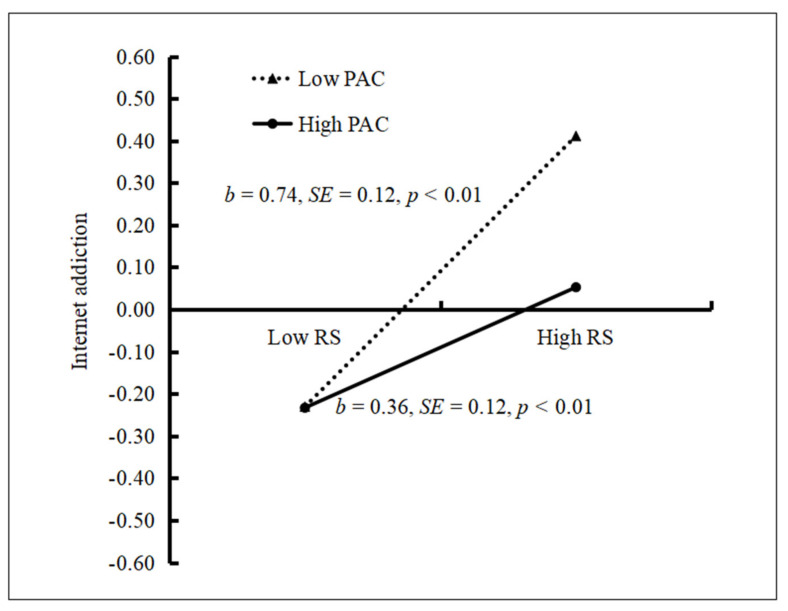
Internet addiction among adolescents as a function of rejection sensitivity and parent–adolescent communication. Note: CV = cybervictimization, PAC = parent–adolescent communication.

Similarly, we conducted a simple slope test to assess the moderating effect of parent–adolescent communication between rejection sensitivity and Internet addiction. It revealed that the positive association between rejection sensitivity and Internet addiction was much stronger for adolescents with lower parent–adolescent communication (*t* = 6.28, *p* < 0.01, 95% CI: 0.51, 0.98) than for those with higher parent–adolescent communication (*t* = 2.71, *p* < 0.01, 95% CI: 0.09, 0.57). See Figure 5.

Lastly, the bias-corrected percentile bootstrap results indicated that the indirect link between cybervictimization and Internet addiction via rejection sensitivity was more significant for adolescents with low parent–adolescent communication (indirect effect = 0.22, 95% CI: 0.11, 0.38) than for those with high parent–adolescent communication (indirect effect = 0.20, 95% CI: 0.06, 0.41). Therefore, the mediating effect of rejection sensitivity between cybervictimization and adolescent Internet addiction was moderated by parent–adolescent communication.

## 4. Discussion

Consistent with our hypotheses, the results showed that cybervictimization is indirectly related to adolescent Internet addiction through rejection sensitivity, and this process is attenuated by positive parent–adolescent communication. This study makes a significant theoretical contribution by advancing our understanding of the association between cybervictimization and adolescent Internet addiction. Moreover, the pathways of our moderated mediation model provide insight into developing preventive interventions for Internet addiction among adolescents.

### 4.1. The Mediating Role of Rejective Sensitivity

Our findings confirmed the hypothesis that the direct association is mediated by rejection sensitivity. This finding supports the rejection sensitivity model [16], which suggests that adolescents who develop rejection sensitivity based on interpersonal stressors are more likely to develop externalizing or internalizing symptoms. Thus, adolescents who internalize the experience of cybervictimization may particularly experience an increase in rejection sensitivity [17,53]. Consequently, they may use the Internet, an anonymous virtual space, to connect with others without pressure and to avoid or reduce their anxiety about future social interactions [54]. 

Additionally, the results for each individual link of our mediation model are noteworthy. First, our findings are consistent with previous studies [55], demonstrating that cybervictimization is positively correlated with rejection sensitivity. It supports the view that interpersonal rejection can lead to anxious or hostile internalized attributions [17]. Adolescents who have experienced cybervictimization tend to attribute future rejections as a fault of their own selves and thus report higher levels of rejection sensitivity. This thought association is consistent with the assumption that cybervictimization is a risk that may be associated with rejection sensitivity. In addition, the second link in the mediation chain also strengthens the previously supported relationship between rejection sensitivity and adolescent Internet addiction [17]. Specifically, adolescents with higher rejection sensitivity are more likely to be addicted to the Internet since they may try to avoid interpersonal stressors or reduce the likelihood of rejection through the Internet. The results of the current study are consistent with those of other studies showing that rejection sensitivity exerts an influence on Internet addiction [17,26].

Finally, according to the rejection sensitivity model [16], adolescents who suffer from interpersonal rejection experience tend to higher rejection sensitivity and then lead to maladaptive behavior [17,18]. Our study was the first to explore that rejection sensitivity performed the mediating function on the relationship between online rejection experience (e.g., cybervictimization) and maladaptive behavior (e.g., Internet addiction), and this relationship could be moderated by parent–adolescent communication. The result extends the rejection sensitivity model: (1) not all adolescents with high rejection sensitivity are equally Internet addicted; (2) not all adolescents with high cybervictimization show equally rejection sensitivity; and (3) Internet addiction and rejection sensitivity are much lower in adolescents with high parent–adolescent communication.

### 4.2. The Moderating Role of Parent–Adolescent Communication

Our results support the hypothesis that parent–adolescent communication moderated the impact of cybervictimization on rejection sensitivity. This protective effect of parent–adolescent communication will weaken as the environmental risks increase. Adolescents sharing higher levels of communication with parents showed better adaptability than those with lower levels, while experiencing a lower severity of cybervictimization. However, no difference was observed when a higher severity of cybervictimization was experienced. This finding supports the self-determination theory [36], which states that adolescents tend to satisfy their psychological needs from their parents, instead of the Internet, after experiencing interpersonal stress, when the parent–adolescent relationship is strong. An explanation for this is that adolescents, who have a close relationship with their parents, tend to share their media experiences with them. Communicating about cybervictimization incidences to parents could help adolescents deal with these experiences in a better manner [37]. However, when the adolescents experienced a higher level of cybervictimization, the protective role of parent–adolescent communication was not significant. One possible explanation is that cybervictimization can increase rejection sensitivity and develop avoidance for interpersonal interactions among the youth. When adolescents are exposed to frequent cybervictimization, avoidance may extend to parental communication and therefore affect this coping mechanism [30,38]. In summary, strong parent–adolescent communication can reduce adolescents’ rejection sensitivity against the negative impact of cybervictimization.

Additionally, we find that parent–adolescent communication moderates the association between rejection sensitivity and Internet addiction. Adolescents with high parent–adolescent communication showed better adaptability in the face of stronger rejection sensitivity. Positive parent–adolescent communication makes adolescents who have experienced bullying feel supported, understood, and loved, and increases parents’ objective understanding of adolescents’ situations. This gradually develops adolescents’ ability to solve problems independently, which is beneficial for promoting their self-esteem and self-protection [30,38]. Thus, adolescents sharing a close relationship with their parents tends to compensate for their social deficits by communicating with them, instead of using the Internet, after experiencing interpersonal stressors. Additionally, rejection sensitivity may cause adolescents to be scared to share their true feelings, due to the fear that they will not be recognized and accepted. To avoid further deterioration of interpersonal relationships, adolescents might choose the Internet to channel their anxiety. However, strong parent–adolescent communication can improve adolescents’ understanding of their immediate emotional reactions and allow them to recognize their emotions, rather than relying on the Internet alone, which will help improve their adaptability. 

The present study found that parent–adolescent communication does not moderate the residual effect of cybervictimization on Internet addiction. Two possible explanations may be considered for this finding. First, adolescents are in a period of rapid self-awareness and may prefer to share their online experiences with their peers [56], which results in parent–adolescent communication not being able to function as a moderator. Second, adolescents with high parent–adolescent communication rarely experienced parental rejection, which results in a poor ability of coping with negative events [57]. Moreover, cybervictimization is a negative interpersonal experience, and adolescents who experience cybervictimization frequently are likely to engage in deviant behavior (e.g., Internet use). In view of the above two reasons, high levels of cybervictimization are particularly harmful to adolescents, which may make the protective effect of parent–adolescent communication insufficient to offset the risk of cyber victimization. It suggests we should recognize the limited protective effect of parent–adolescent communication and that targeted preventive interventions should be implemented for adolescents who are exposed to cybervictimization.

### 4.3. Limitations and Future Directions

Several limitations must be considered while interpreting the results of this study. First, although self-reported assessments of Internet addiction in adolescents are widespread, future studies could try to collect data from different sources (parents, teachers, peers, etc.) to verify the reliability of the results. Second, there may be other moderators (e.g., teacher–student relationships) that are central to the moderation process [18]. Third, this study is a cross-sectional survey, so the results should be interpreted with caution in terms of causality. Future studies should conduct longitudinal or experimental research to confirm the causal relationships among these variables. Additionally, the present findings are limited to adolescents from the Guangdong Province, China. Future studies should examine whether the findings can be generalized to other developmental stages, regions, and cultures. Finally, further studies should explore the prevalence of the issue of “which adolescent victims reject ‘internet’ and who are addicts?” This may provide us with a comprehensive understanding of the impact of cybervictimization on adolescents’ development. 

## 5. Conclusions

The current study extends the rejection sensitivity model [16] and the self-determination theory [36] to the study of Internet addiction by testing the mediating and moderating role of rejection sensitivity and parent–adolescent communication, respectively. The current moderated mediation model enriches our understanding of the mechanisms underlying the relationship between cybervictimization and Internet addiction among Chinese adolescents. Our findings indicate that cybervictimization can indirectly impact adolescent Internet addiction through rejection sensitivity. Moreover, cybervictimization does not act as a single key to Internet addiction but rather interacts with other factors, such as parent–adolescent communication, to contribute to adolescent Internet addiction.

### Implications for Practice 

Firstly, this study found that cybervictimization is an important risk factor for adolescent Internet addiction. It is critical for parents and educators to early identify youth who have suffered from cybervictimization and provide timely diversion to them. Secondly, rejection sensitivity can significantly mediate the positive link between cybervictimization and adolescent Internet addiction. Therefore, reducing adolescent’s rejection sensitivity can help to prevent adolescent Internet addiction. An effective strategy is to increase the social skills of individuals with high rejection sensitivity to enhance their social confidence, so that they will expect to be accepted rather than rejected [58]. Thirdly, the finding indicated that parent–adolescent communication buffers the risk effect of cybervictimization on adolescent Internet addiction via rejection sensitivity. Therefore, parents should create a supportive family environment to promote positive communication and interaction with their children.

## Figures and Tables

**Table 1:** Demographic information about participants.

Variables	*n*	*%*
Gender		
Male	485	48.20
Female	521	51.80
Age		
12–12.99 years	423	42.05%
13–13.99 years	433	43.04%
14–14.99 years	150	14.91%

**Table 2:** Descriptive statistics and correlation for all variables.

Variables	1	2	3	4	5	6	7
1.Gender	1.00						
2.Age	0.06 *	1.00					
3.Impulsivity	0.00	−0.08 *	1.00				
4.CV	0.02	0.00	0.21 **	1.00			
5.PAC	−0.02	−0.14 **	−0.29 **	−0.14 **	1.00		
6.RS	−0.20 **	0.01	0.31 **	0.23 **	−0.13 **	1.00	
7.IA	0.07 *	0.02	0.35 **	0.30 **	−0.20 **	0.29 **	1.00
α	—	—	0.82	0.82	0.68	0.86	0.74
Mean	—	13.16	2.12	1.13	2.26	3.04	1.18
SD	—	0.67	0.40	0.20	0.53	0.43	1.25

Note: Gender and age were dummy coded such that 1= male, 0 = female. CV = Cybervictimization, RS = rejection sensitivity, PAC= parent–adolescent communication, IA = Internet addiction. * *p* < 0.05, ** *p* < 0.01.

## Data Availability

The data presented in this study are available on request from the corresponding authors (C.Y.).
